# Genome-Wide Analysis of the Serine Carboxypeptidase-like (SCPL) Protein Family of Bitter Gourd and Functional Validation of *McSCPL*22 in *Fusarium oxysporum* f. sp. *Momordicae* (FOM) Resistance

**DOI:** 10.3390/ijms252111816

**Published:** 2024-11-03

**Authors:** Feng Guan, Xuetong Yang, Bo Shi, Kai Wang, Jingyun Zhang, Yuanyuan Xie, Xinjian Wan

**Affiliations:** 1 Institute of Vegetables and Flowers, Jiangxi Academy of Agricultural Sciences, Nanchang 330200, China; guanfeng_0813@163.com (F.G.); yangxuetong2023@163.com (X.Y.); shibo_jiangxi@163.com (B.S.); wangkai2023jx@163.com (K.W.); zhangjingyun0108@126.com (J.Z.); m18073933758@163.com (Y.X.); 2 Jiangxi Key Laboratory of Horticultural Crops (Fruit, Vegetable & Tea) Breeding, Jiangxi Academy of Agricultural Sciences, Nanchang 330200, China; 3 Jiangxi Engineering Research Center of Vegetable Molecular Breeding, Jiangxi Academy of Agricultural Sciences, Nanchang 330200, China

**Keywords:** bitter gourd, serine carboxypeptidase-like family, *Fusarium oxysporum* f. sp. *momordicae*, expression analysis

## Abstract

Bitter gourd is increasingly being recognized for its value as a vegetable and medicinal use, but the molecular mechanisms of pathogen resistance remain relatively poorly understood. The serine carboxypeptidase-like (SCPL) protein family plays a key role in plant growth, pathogen defense, and so on. However, a comprehensive identification and functional characterization of the *SCPL* gene family has yet to be conducted in bitter melon. In this study, 32 *SCPL* genes were identified in bitter gourd and divided into three classes. The number of *SCPL* genes contained in the three clusters was 7, 7, and 18, respectively. Most *SCPL* gene promoters contain *cis*-acting elements with light, hormone, and stress responses. The RNA sequencing data showed that the expression of several *SCPL* genes changed significantly after pathogen infection. In particular, expression of the *McSCPL4*, *10*, *17*, *22*, and *25* genes increased substantially in the resistant varieties after infection, and their expression levels were higher than those in the susceptible varieties. These results suggested that genes such as *McSCPL4*, *10*, *17*, *22*, and *25* may play a significant role in conferring resistance to fungal infections. Moreover, the expression levels of the *McSCPL10*, *17*, *22*, *23,* and *25* genes were likewise significantly changed after being induced by salicylic acid (SA) and jasmonic acid (JA). In situ hybridization showed that *McSCPL22* was expressed in the vascular tissues of infected plants, which largely overlapped with the location of *Fusarium oxysporum* f. sp. *Momordicae* (FOM) infection and the site of hydrogen peroxide production. Our results showed that *McSCPL22* may be involved in the regulation of the SA and JA pathways and enhance resistance to FOM in bitter gourd plants. This is the first study to perform *SCPL* gene family analysis in bitter gourd. *McSCPL22* may have the potential to enhance FOM resistance in bitter gourd, and further investigation into its function is warranted. The results of this study may enhance the yield and molecular breeding of bitter gourd.

## 1. Introduction

*Momordica charantia* L. is a member of the *Cucurbitaceae* family; we commonly call it bitter gourd or bitter melon [1]. Bitter gourd has important nutritional and medical value and is used for medicinal purposes such as treating cancer, inflammation, and high cholesterol [2,3]. The production of bitter gourd is affected by some insects, such as the gourd fruit fly [4], and is also susceptible to various fungal infections, such as *Fusarium oxysporum* f. sp. *momordicae* (FOM), which results in reduced yields [5,6]. To reduce the damage caused by pests and diseases, researchers aim to identify genetic resources with pest and disease resistance. In our previous studies, the resistant and susceptible varieties of bitter gourd were researched by transcriptome and metabolome methods for screening the candidate genes [7,8].

In plants, serine carboxypeptidase-like proteins (SCPLs) belong to the serine carboxypeptidase family. They have a highly conserved *α*/*β* hydrolase fold, which is characterized by a central *β*-sheet surrounded by α-helices on either side [9,10]. SCPLs exhibit acyltransferase activity rather than peptidase activity. Typically, SCPLs contain a conserved amino acid structure of serine, aspartate, and histidine (Ser-Asp-His) with a typical PF00450 domain [11]. Previous research has demonstrated that SCPLs have complex roles in plant growth and resistance to various stresses [12,13,14,15].

In particular, SCPLs were found to play a significant role in plant defense mechanisms. In soybeans, at least six GmSCPLs were found to respond to various adverse stresses [16]. In cotton, GhSCPL42 may increase the resistance of cotton to Verticillium wilt through the salicylic acid (SA) and jasmonic acid (JA) pathways [17]. The rice OsSCPL1 gene was observed to regulate the defense responses of the plant against the bacterial pathogen *Pseudomonas syringae* pv. tomato and the fungal pathogen *Alternaria brassicicola* [14]. Oat SCPL1 was found to be essential for antimicrobial synthesis and disease resistance [15].

SCPLs are widely distributed in various plants, and SCPL families have been successfully characterized in several species, with results showing that there may be 54 SCPL family members in *Arabidopsis thaliana* [18,19], 71 members in rice (*Oryza sativa* L. ssp. *japonica*) [20], 57 members in poplar (*Populus tomentosa*) [21], 47 members in the tea plant (*Camellia sinensis*) [22], 209 members in wheat (*Triticum aestivum*) [23], 117 in *Brassica napus* [24], and 73 members in soybean (*Glycine max*) [16]. Moreover, Fu et al. identified 891 SCPL genes from 14 species [25]. Currently, the *SCPL* gene family has not been researched in bitter gourd.

In this study, genome-wide characterization of the SCPL gene family was performed using the available bitter gourd reference genome. The expression patterns of SCPLs were investigated using available transcriptome data and were detected in the treatment plant with SA and JA. Subsequently, genes that may be related to fungal resistance were screened. These results will provide a reference for further systematic studies on the functions of the SCPL genes in bitter gourd and additional genetic resources to enrich resistance breeding in bitter gourd.

## 2. Results

### 2.1. Identification of the Bitter Gourd SCPL Gene Family

We identified 32 *McSCPL* genes in bitter gourd and confirmed them in the CDD and SMART databases. All *McSCPLs* can be found in conserved domains in the SMART database. The 32 *McSCPL* genes were named McSCPL1–32 (Table 1). The coding protein length ranged from 268 to 1457 amino acids. The molecular weight ranged from 31.0 to 161.6 kDa, and their isoelectric point was 4.85–8.8. Among them, 28 proteins were localized in the vacuole, while McSCPL26 was found in the peroxisome. Additionally, McSCPL9, McSCPL11, and McSCPL12 were observed in both the vacuole and peroxisome (Table 1).

### 2.2. Evolutionary Relationship of SCPL Genes

**Figure 1:**
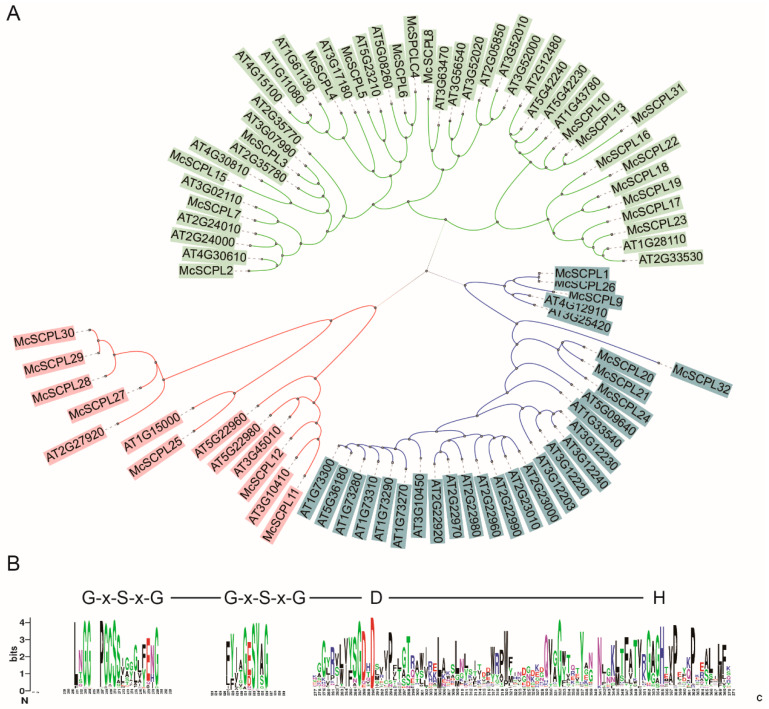
Evolutionary relationships of SCPL proteins from *M. charantia* and *A. thaliana.* (**A**) The evolutionary history analysis. (**B**) The main motif logo of SCPL. The characters show the consensus sequence. G, glycine; S, serine; x, any amino acid.

The predicted SCPL protein sequences from *M. charantia* and *A. thaliana* were used to construct a phylogenetic tree. The SCPL proteins clustered into three subgroups: green, blue, and red (Figure 1A). There were 18, 7, and 7 SCPL proteins in the green, blue, and red subgroups of *M. charantia*, respectively. In the green, blue, and red subgroups, there were 26, 22, and 6 SCPL proteins of *A. thaliana*, respectively (Figure 1A). The SCPL genes in the green and red subgroups of *M. charantia* were similar to those of *A. thaliana*, but in the blue subgroup, *M. charantia* had only one-third of the number of genes that *A. thaliana* had.

The multiple sequence alignment shows that McSCPLs and AtSCPLs have a typical Ser-Asp-His conserved domain and two typical G-x-S-x-G motifs. The Ser was surrounded by a Gly structure (Figure 1B).

### 2.3. Evolutionary Tree, Sequence Structure Analysis of SCPL Genes

**Figure 2:**
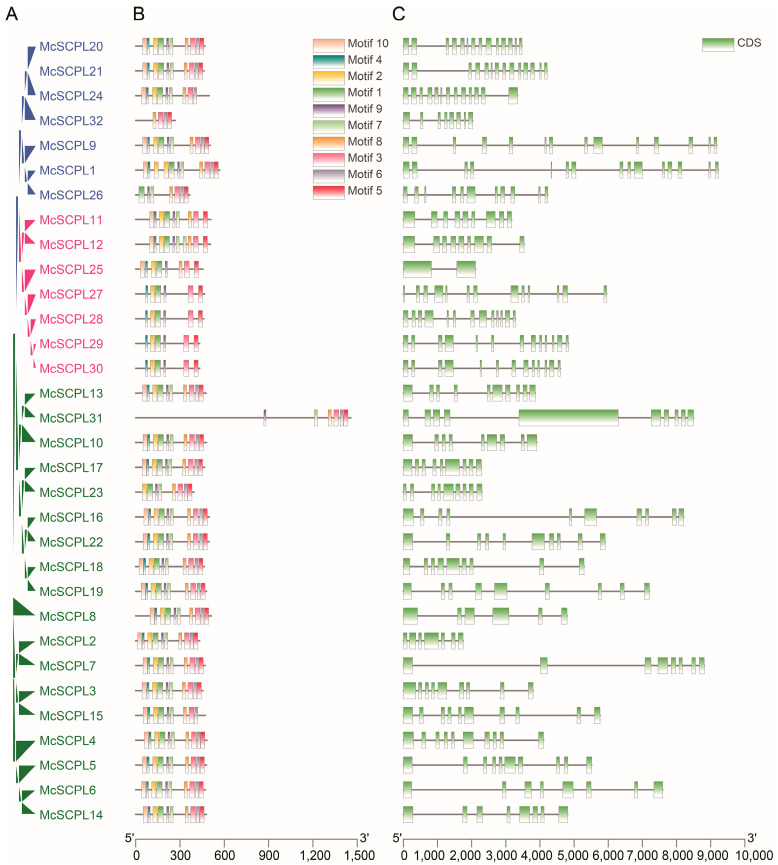
Gene structure of McSCPLs. (**A**) Phylogenetic tree of *McSCPL*-encoding proteins. (**B**) The conserved motif distribution in the McSCPL protein sequence. The character sequence of each motif is shown in Supplementary Figure S1. (**C**) Genomic structure diagram of McSCPLs in bitter gourd. Exons are represented with green boxes; introns are shown with lines.

A phylogenetic tree of McSCPL proteins showed that all members were divided into three subgroups, which were consistent with the findings in other known plants (Figure 2A). Among them, all members contained motif 3 and motif 5, but the red subgroup did not contain motif 6, and the blue and green subgroups exhibited similar motif characteristics (Figure 2B, Supplementary Figure S1). The green subgroup contains 8–10 exons, the red subgroup members have 9–13 exons, except for McSCPL25, which only has two exons, and the blue subgroup members have 8–15 exons, which have the greatest range of exon numbers (Figure 2C).

### 2.4. The Cis-Acting Elements of the SCPL Genes Promoter

**Figure 3:**
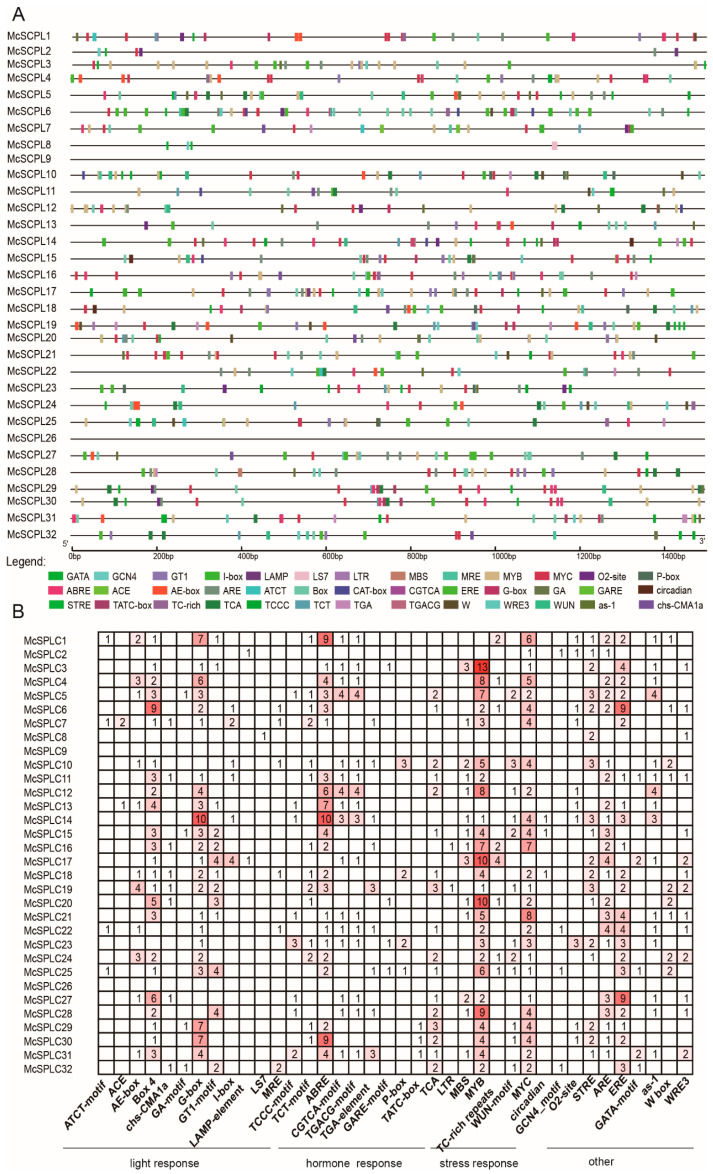
*cis*-element distribution of *McSCPL* genes. (**A**) Location of the *cis*-elements. (**B**) Quantitative analysis of the *cis*-elements.

To predict the possible regulatory factors of the McSCPL family members, the upstream promoter sequences 1500 bp before the start codon of the McSCPL family members were analyzed. The McSCPL9 and McSCPL26 promotor sequences cannot be obtained because the genome sequences are incomplete. The results revealed that the promoters of these genes are mainly *cis*-elements, including light, hormone, and stress responses. The promoters of most of the gene family members contain light response-related elements, such as Box-4 and G-box; methyl jasmonate-related *cis*-elements CGTCA-motifs and TGACG-motifs; and SA-related *cis*-element TCA; abscisic acid-related *cis*-element ABRE; and defense and stress response-related *cis*-elements of MYB binding (Figure 3A). In addition, the specific type and quantity of *cis*-elements present within each gene promoter exhibited variability. This suggests that most of these family members may be regulated by abscisic acid, methyl jasmonate, and SA and are closely associated with biotic and abiotic stress responses. These genes may be regulated by auxin (McSCPL7, McSCPL10, McSCPL14, McSCPL16, McSCPL19, McSCPL22, McSCPL25, McSCPL28, and McSCPL31 containing the TGA-element) or gibberellin (eight genes contained at least one *cis*-element of the GARE-motif, or P-box, or TATC-box) (Figure 3B).

### 2.5. Tissue-Specific Expression Profiles of SCPL Genes

**Figure 4:**
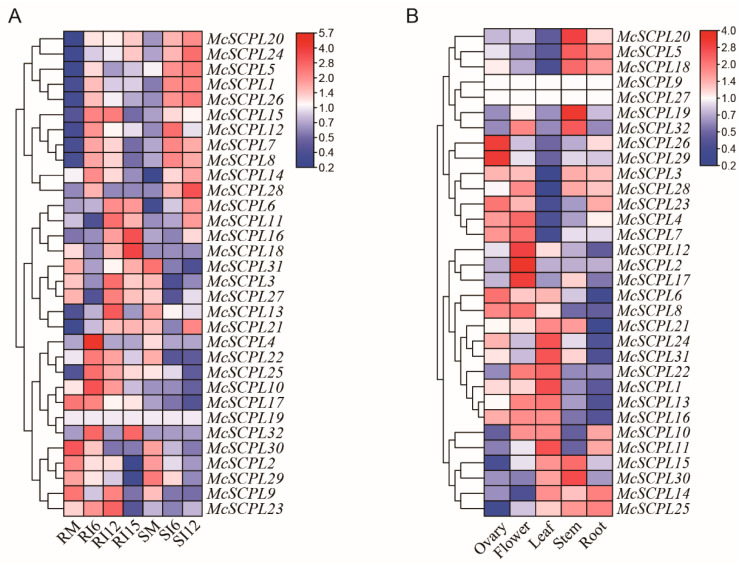
Expression pattern of *McSCPL*s. (**A**) Expression patterns after being treated by FOM. RM, mock root tissues of the resistant variety (*BK0604*); SM, mock root tissues of the susceptible variety (*LK0901*). Samples of the inoculated roots were collected at 6 days (RI6 and SI6), 12 days (RI12 and SI12), and 15 days (RI15). (**B**) Expression patterns in tissues of bitter gourd cultivar *Dali-11*. The data are log2-transformed and represented by the color bar.

The expression patterns of *McSCPL* genes were calculated based on previous report data [7]. The expression of the *McSCPL1*, *McSCPL5*, *McSCPL7*, *McSCPL8*, *McSCPL12*, *McSCPL14*, *McSCPL15*, *McSCPL20*, *McSCPL24*, *McSCPL26*, and *McSCPL28* genes showed a trend of upregulation after inoculation with FOM both in resistant and susceptible varieties. The expression of *McSCPL3*, *McSCPL6*, *McSCPL11*, *McSCPL16*, *McSCPL18*, *McSCPL27*, and *McSCPL31* genes showed a trend of downregulation after inoculation with FOM, both in resistant and susceptible varieties. A comprehensive evaluation of the expression level of the *McSCPL2*, *McSCPL4*, *McSCPL9*, *McSCPL10*, *McSCPL17*, *McSCPL22*, *McSCPL23*, *McSCPL25*, *McSCPL29*, *McSCPL30*, and *McSCPL32* genes revealed that they were higher in the resistant variety than in the susceptible variety at multiple time points in both mock and treated samples (Figure 4A).

In the tissues, *McSCPL26* and *McSCPL29* showed higher expression in the ovary tissue; *McSCPL2, McSCPL12,* and *McSCPL17* were highly expressed in the flowers; and *McSCPL5*, *McSCPL9*, *McSCPL18-20,* and *McSCPL32* were highly expressed in the stem; *McSCPL1, McSCPL10*, *McSCPL11*, *McSCPL13-16*, *McSCPL21*, *McSCPL22*, *McSCPL24*, *McSCPL25*, *McSCPL30*, and *McSCPL31* were highly expressed in the leaf (Figure 4B).

### 2.6. Expression Analysis of McSCPLs Response to SA and JA Treatment

**Figure 5:**
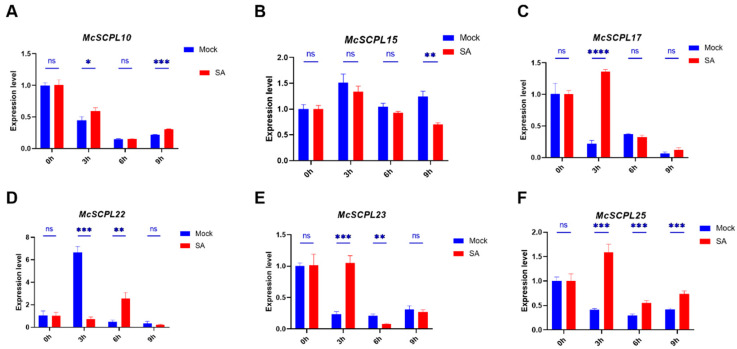
Expression of *McSCPLs* in response to SA treatment. (**A**) Expression level of *McSCPL10.* (**B**) Expression level of *McSCPL15.* (**C**) Expression level of *McSCPL17.* (**D**) Expression level of *McSCPL22.* (**E**) Expression level of *McSCPL23.* (**F**) Expression level of *McSCPL25.* The bar showed standard errors (SE). ns: no significance; * *p* < 0.05; ** *p* < 0.01; *** *p* < 0.001; **** *p* < 0.0001 (Student’s *t*-test).

SA and JA have important functions in disease resistance. To confirm that *McSCPL*s are regulated by SA and JA, the results demonstrated that *MCSCPL10* and *MCSCPL15* had a similar expression change to the control after SA treatment. The expression of *McSCPL10* was higher than the control group at three and nine hours, and the expression of *McSCPL15* was lower than the control group at nine hours (Figure 5A,B). SA promotes the expression of *McSCPL17*, *McSCPL23*, and *McSCPL25*. Their expression levels were significantly higher at three hours after treatment than in the control group (Figure 5C,E,F). The expression of *McSCPL22* was markedly elevated in the control group three hours after treatment, subsequently declining (Figure 5D), indicating that SA affects the expression level of *McSPLC22*.

**Figure 6:**
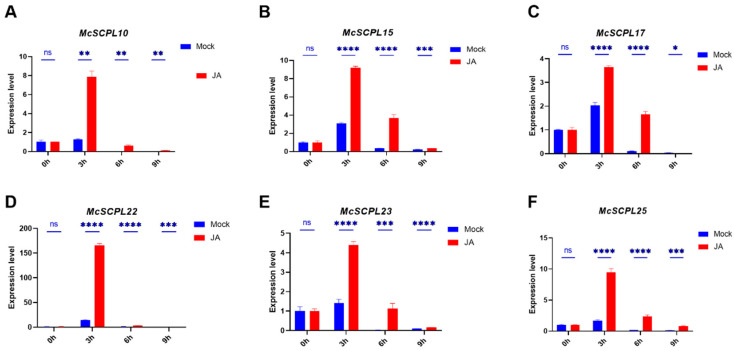
Analysis of the expression of certain *McSCPLs* in response to JA treatment. (**A**) Expression level of *McSCPL10.* (**B**) Expression level of *McSCPL15.* (**C**) Expression level of *McSCPL17.* (**D**) Expression level of *McSCPL22.* (**E**) Expression level of *McSCPL23.* (**F**) Expression level of *McSCPL25.* The bar showed standard errors (SE). ns: non significance; * *p* < 0.05; ** *p* < 0.01; *** *p* < 0.001; **** *p* < 0.0001 (Student’s *t*-test).

After JA treatment, the expression level of *McSCPL10*, *15*, *17*, *22*, *23*, and *25* was elevated at three hours. This elevation was followed by a gradual decrease (Figure 6A–F). This suggests that JA may promote the expression of multiple *McSCPLs*.

### 2.7. FOM Infection Promoted the Accumulation of Hydrogen Peroxide

**Figure 7:**
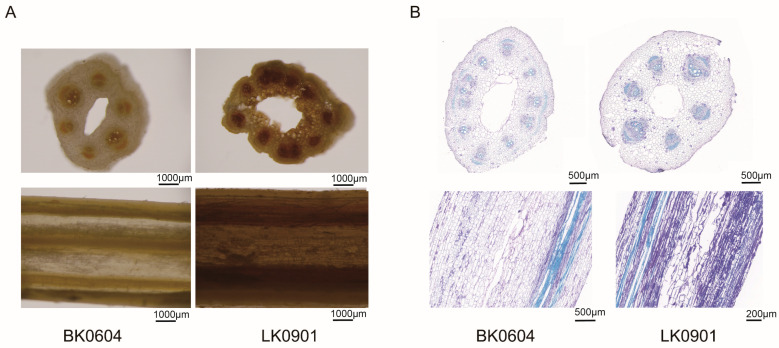
Detection of hydrogen peroxide accumulation. (**A**) Staining results of hydrogen peroxide in the stems after FOM infection. (**B**) Methylamine blue staining of stems. BK0604, resistant variety; LK0901, susceptible variety.

In plants, fungal infection frequently results in the generation of reactive oxygen species. We examined the infected bitter gourd stems and showed that hydrogen peroxide (brown parts) was produced in the stems of both resistant (BK0604) and susceptible (LK0901) varieties (Figure 7A). However, the accumulation of hydrogen peroxide in the susceptible varieties was higher than in the resistant varieties, which suggests that resistant varieties have an inhibitory effect on the accumulation of hydrogen peroxide. The staining of the sites where hydrogen peroxide was produced mainly focused on the locations where the vascular bundles were located, including the xylem and phloem (Figure 7B).

### 2.8. McSCPL22 May Respond to FOM Infection

**Figure 8:**
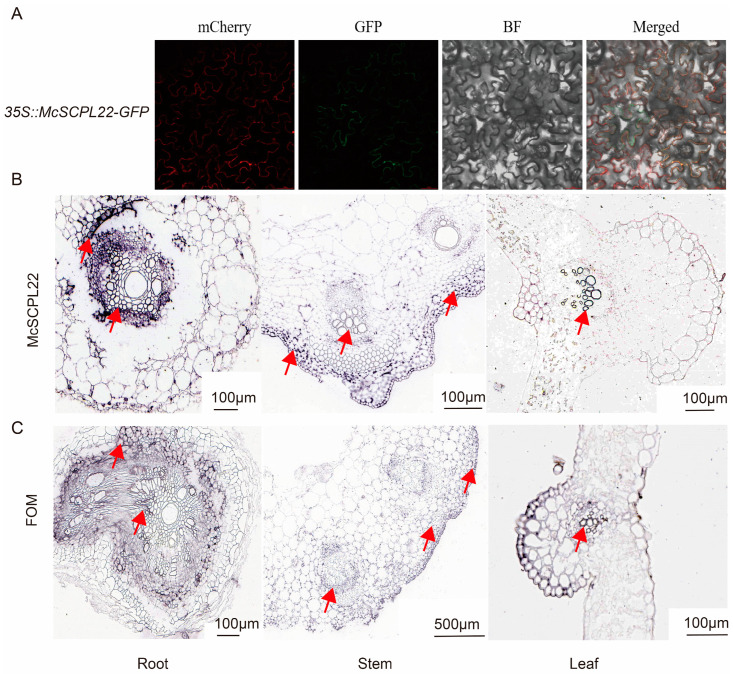
The expression of McSCPL22. (**A**) Subcellular localization of McSCPL22. (**B**) In situ hybridization of the *McSCPL22* gene. (**C**) In situ hybridization of the FOM probe. The red arrow shows the expression site.

From the quantitative PCR results, it can be seen that there is a large difference in the expression of *McSCPL22* at 3 h after SA and JA treatments compared with the control group. Subcellular localization studies revealed that *McSCPL22* is mainly localized to the cell membrane (Figure 8A).

By in situ hybridization, we found that after FOM inoculation, *McSCPL22* was mainly detected in the vascular bundle portion of the plant, in addition to some expression in the cortical thin-walled tissue of the stem (Figure 8B). Interestingly, the hybridization of the infected tissues by the nucleic acid probe for FOM showed that the distribution location of FOM was also mainly concentrated in the vascular bundle part of the plant, which was the same site of McSCPL22 expression (Figure 8C).

## 3. Discussion

Bitter gourd is increasingly becoming a valuable food and medical resource, but it is relatively understudied for stress tolerance, with members of the *SCPL* gene family showing tolerance to biotic and abiotic stresses in several species [10,15,23,26,27]. The *SCPL* gene family is characterized and reported in multiple species [16,17,18,20,21,22,23,24,25]; however, this is the first time that the *SCPL* gene family has been identified in bitter gourd. In this study, we searched the bitter gourd genome with *A. thaliana* SCPL family members using BLASTP and simultaneously used HMMER based on the *SCPL* gene model (PF00450). We found that the 32 members of the bitter gourd theoretical isoelectric point varied widely (4.85–8.8), but most of the isoelectric points were less than 7 (approximately 65%) and shared similar features with other species.

As we know, *Arabidopsis* has 54 SCPL family members, which is close to twice the number of family members in bitter gourd, but in terms of the evolutionary relationship, we found that the number of *SCPLs* in the red subfamily was similar in bitter gourd and *Arabidopsis*, which are 7 and 6, respectively. In the blue cluster subfamily, the number of members in bitter gourd is 7, but the number of members in *Arabidopsis* is 22, which is close to three times that in bitter gourd. In *Arabidopsis*, the functions of the members of this subfamily are relatively well studied, such as At5G09640 (sinapoylglucose choline sinapoyltransferases) [28], AT2G22990 (sinapoylglucose malate sinapoyltransferase) [29], AT2G23000 (sinapoylglucose anthocyanin sinapoyltransferase) [30], and AT2G23010 and AT2G22980 (sinapoylglucose sinapoyltransferase) [30]. In addition, the functions of the members of this subfamily in *Arabidopsis* mainly regulated the formation of a variety of secondary metabolites, whereas the relatively small number of members of this subfamily in bitter gourd may imply that the corresponding secondary metabolites may be lacking in bitter gourd. In addition, Mugford et al. found that AsSCPL1, a gene with functions in the synthesis of antimicrobial compounds and disease resistance, belongs to the blue cluster subfamily. This finding suggests that members of this subfamily may have evolved resistance-related functions [15]. The green cluster subfamily of bitter gourd has 18 SCPL members, whereas *Arabidopsis* has 26 members; the ratio of subfamily genes is similar to the overall number.

Because of the rich functionality of SCPL family members, the results of analyzing their upstream promoter elements revealed that most of the members contain multiple types of elements, including light, hormone, and stress responses (Figure 3). These results suggest that these members are related to the corresponding regulatory pathways. Combined with previously reported transcriptome data [7], it was shown that the expression of most of the genes changed significantly after FOM infection, especially in the resistant varieties, where the expression level was significantly elevated, such as *McSCPL4*, *McSCPL22*, *McSCPL25*, and *McSCPL10* (Figure 4A). We also found several hormone-related regulatory elements in their promoter sequences. In a previous report, cotton GhSCPL42 improved FOM resistance through the SA and JA pathways [15]. We found significant changes in the expression level of multiple SCPL members in bitter gourd after SA and JA treatment (Figure 5 and Figure 6). These results indicate that *McSCPL10*, *McSCPL22*, and *McSCPL25* might be involved in bitter gourd resistance to FOM via the SA and JA regulatory pathways. Interestingly, *McSCPL22* was significantly downregulated after treatment with SA and upregulated after treatment with JA, which varied dramatically and could serve as a genetic resource to consider in subsequent studies.

To preliminarily determine the function of *McSCPL22*, we found it mainly localized to the cell membrane, which differed from the predicted result in the vacuole (Figure 8A). Following inoculation with FOM, the susceptible varieties produced more hydrogen peroxide, mainly concentrated within the vascular bundles (Figure 7). This observation is similar to the FOM distribution after infection (Figure 8C). Interestingly, we also found that the expression of *McSCPL22* in the infected susceptible varieties was also concentrated in the vascular bundles of the plants (Figure 8B). This result suggests that the site fungal infection and the expression of the *McSCPL22* gene may overlap. The expression level of *McSCPL22* was markedly increased in the resistant varieties after FOM inoculation, suggesting a potential relation between the stress response and elevated resistance of bitter gourd.

This study identified and reported the SCPL gene family of bitter melon for the first time. Furthermore, the evolutionary relationships, gene structures, conserved motif features, and expression patterns of the SCPL family in bitter melon were then systematically analyzed and compared. It was found that most of the *McSCPLs* might be associated with light, hormone, and stress responses. The results analyzed by gene expression patterns and in situ hybridization showed that *McSCPL22* may respond to FOM infection in bitter melon via the SA and JA regulatory pathways. The results presented herein provide a foundation for further investigation into the functions of McSCPLs and the molecular mechanism of *McSCPL22* in fungal resistance, as well as the potential use of *McSCPL22* in the breeding of highly resistant bitter melon varieties.

## 4. Methods

### 4.1. Plant Material

This research used two bitter gourd varieties; BK0604 was highly resistant to Fusarium wilt and selected and bred from a local variety in Jiangxi Province by the study team. LK0901 was highly susceptible to Fusarium wilt collected from Taiwan Province in 2004 and was bred by our team [7]. Bitter gourd seeds were soaked in water for 17 h and germinated in a controlled environment at 32 °C. After germination, the seedlings were transplanted into pots with the seedling substrate (Pindstrup, Ryomgård, Danmark) for further experimentation.

### 4.2. Identification of SCPL Genes

The genome data of bitter gourd were downloaded from the National Center for Biotechnology Information (NCBI) database (Assembly version: GCF_001995035.1; PRJNA397875) [31]. *Arabidopsis SCPL* gene data were acquired from the TAIR database (https://www.arabidopsis.org, accessed on 30 September 2023). The *SCPL* gene model PF00450 was obtained from the Pfam website (http://pfam.xfam.org, accessed on 30 September 2023). The PF00450 data were queried in encoding protein sequence data of bitter gourd using HMMER software (v3.0). The results were further verified through Pfam, NCBI CDD, and SMART databases [32,33,34,35].

### 4.3. Phylogenetic Analysis of the SCPL Gene Family

The ClustalW program facilitated multiple alignments of the SCPL protein sequences from *M. charantia* and *Arabidopsis*. A phylogenetic tree was constructed utilizing the MEGA7 software (v7) through the neighbor-joining method, employing the Poisson correction model with 1000 bootstrap test replications [36].

### 4.4. Structure Analysis of the SCPL Gene Family

The gene structure file was submitted to the GSDS 2.0 server to obtain the diagram [37]. The chromosome location of the *McSCPL* genes was retrieved from *M. charantia* genome data. We used the MEME website (https://meme-suite.org/meme/, accessed on 10 July 2023) to identify conserved protein motifs using default parameters. The Plant-mPLoc website (http://www.csbio.sjtu.edu.cn/bioinf/Cell-PLoc-2/, accessed on 20 June 2023) was used to predict the subcellular location of McSCPLs. The molecular weight and isoelectric point of the McSCPL proteins were predicted using Expasy. Then, the cis-acting elements of the 1500 bp promoter sequences of each *McSCPL* gene were analyzed via the PlantCARE website (http://bioinformatics.psb.ugent.be/webtools/plantcare/html/ , accessed on 10 July 2023).

### 4.5. Expression Pattern of SCPL Genes Based on RNA Sequencing Data

RNA sequencing (RNA-seq) data of the tissue samples (ovary, male, flower, leaf, stem, root) were obtained from the website at http://www.ncbi.nlm.nih.gov/sra/ through the accession numbers SRR13308550–SRR13308554 (PRJNA687997) (accessed on 5 October 2023). The data related to the fungal infection assays were obtained from the following source: http://www.ncbi.nlm.nih.gov/bioproject/PRJNA825666 (accessed on 5 October 2023) [7]. The fragments per kilobase of transcript per million mapped reads (FPKM) values for each gene in all samples were calculated according to our previous report [7].

### 4.6. Fungal Infection Assay and Treatment with SA and JA

The FOM strain was collected from the experimental base of the Institute of Vegetables and Flowers at Jiangxi Academy of Agricultural Sciences [38]. The FOM was cultured and collected according to previous methods [7]. Liquid potato dextrose agar medium at 25 °C with shaking at 150 rpm for 5–7 days. The conidia with 1 × 10^6^ CFU/mL concentration were used to infect the bitter gourd root. Bitter gourd seedlings were washed and dipped in spore suspension. These seedlings were then replanted into a sterile growing substrate and positioned in an artificial climate chamber set at 28 °C with a 12/12 h light/dark cycle and 70% relative humidity [8]. Seedling leaves were sprayed with SA (1.5 mmol/L) and JA (100 µmol/L) on the leaves at 0, 3, 6, and 9 days after inoculation, respectively [7,8,14]. The control group was treated with distilled water. Then, samples were collected at 2-day intervals after the treatments. Three independent experiments and at least six plants for each experiment were performed.

### 4.7. Gene Expression Analysis by Quantitative Real-Time Polymerase Chain Reaction (qRT-PCR)

Total RNA was extracted, and cDNA was synthesized using RNA Isolation Reagent and cDNA Synthesis Kit (GeneCopoeia, China), respectively. The primers were designed on the Primer3 website (Supplementary Table S1). *McActin7* was used as the internal control. qRT-PCR was performed according to previous methods [7]. The 2^−△△Ct^ method was used to assess gene expression level. Three independent experiments and three technical replicates for each experiment were performed. The obtained data were subjected to unpaired two-tailed Student’s t-tests using GraphPad Prism software (version 8).

### 4.8. Subcellular Localization of the McSCPL22 Protein

McSCPL22 was synthesized into the pR101-35S-GFP vector, then transformed into *Agrobacterium tumefaciens* strain GV3101. *Agrobacterium* containing pCambia1300-35S-PM-mcherry and the pR101-35S-McSCPL22-GFP vector were injected into the 2–4-week-old *Nicotiana benthamiana* leaves. The plants were darkened for 2–3 days. The leaves were cut to observe the fluorescence using a confocal microscope (Leica SP8, Leica, Japan).

### 4.9. Analysis of Hydrogen Peroxide Accumulation

The plant stems were collected after 12 days of the fungal infection assay. H_2_O_2_ content was detected using 3,3′-diaminobenzidine (DAB) staining (Servicebio, Wuhan, China). The stems of BK0604 (R) and LK0901 (S) plants were dipped in 1 mg/mL DAB solution (pH 3.8) for 24 h. Thereafter, the stem sections were boiled in 95% ethanol for 10 min, and the image was captured using Nikon Eclipse CI microscope (Nikon, Japan) [39].

### 4.10. Toluidine Blue Staining

The collected stems were cut into sections and placed into a dewaxing transparent liquid (20 min) (Servicebio, Wuhan, China), anhydrous ethanol (5 min), and 75% alcohol (5 min). The plant tissue sections were stained with toluidine blue dye for approximately 2 min, according to the depth of tissue coloring, and then dried in an oven at 60 °C after washing with water. Finally, the sections were treated with xylene and imaged by Nikon Eclipse E100 (Nikon, Japan) [40,41].

### 4.11. In Situ Hybridization Experiment

This experiment was performed by using standard in situ hybridization and NBT histochemistry techniques [42]. In brief, the tissues of LK0901(S) were placed in situ hybridization fixative (Servicebio, Wuhan, China) overnight at 4 °C, then sliced into 6 μm sections using a microtome. After dewaxing and dehydration, the hybridization solution containing the probes was added as previously described (Supplementary Table S1) [43,44]. Imaging was performed using a Nikon Eclipse CI microscope (Nikon, Japan).

## Figures and Tables

**Table 1:** List of bitter gourd SCPL genes.

NCBI_ID	Renamed	CDD	SMART	Length	MW (Da)	pI	Subcellular Localization
LOC111013428	McSCPL1	+	+	569	63,021.2	4.87	Vac
LOC111007166	McSCPL2	+	+	433	48,972.9	8.8	Vac
LOC111010259	McSCPL3	+	+	456	51,938.3	7.22	Vac
LOC111010625	McSCPL4	+	+	482	54,264.1	5.53	Vac
LOC111014585	McSCPL5	+	+	480	54,343.4	8.31	Vac
LOC111020681	McSCPL6	+	+	472	52,963.4	7.09	Vac
LOC111022214	McSCPL7	+	+	470	52,931.4	8.41	Vac
LOC111014800	McSCPL8	+	+	512	57,242.1	7.25	Vac
LOC111018253	McSCPL9	+	+	505	56,220.9	6.32	Pero
LOC111011999	McSCPL10	+	+	480	53,752.8	6.24	Vac
LOC111006031	McSCPL11	+	+	509	57,031.1	5.2	Pero, Vac
LOC111020491	McSCPL12	+	+	506	56,443.7	5.44	Pero, Vac
LOC111021720	McSCPL13	+	+	477	53,267.2	6.94	Vac
LOC111020263	McSCPL14	+	+	479	54,015.8	6.79	Vac
LOC111024700	McSCPL15	+	+	471	53,692.9	5.09	Vac
LOC111024586	McSCPL16	+	+	498	55,155.1	6.67	Vac
LOC111007100	McSCPL17	+	+	467	52,517.2	7.05	Vac
LOC111023593	McSCPL18	+	+	465	51,808.3	6.7	Vac
LOC111021347	McSCPL19	+	+	480	53,215.9	6.79	Vac
LOC111025862	McSCPL20		+	470	53,137.9	6.1	Vac
LOC111025866	McSCPL21		+	463	51,972.6	6.98	Vac
LOC111019830	McSCPL22	+	+	498	55,001.1	8.58	Vac
LOC111015180	McSCPL23	+	+	395	44,625.3	6.85	Vac
LOC111025759	McSCPL24		+	498	56,128.5	6.97	Vac
LOC111018332	McSCPL25		+	456	50,580.4	6.42	Vac
LOC111013427	McSCPL26	+	+	366	40,877.8	4.85	Pero
LOC111020504	McSCPL27		+	467	52,041.8	6.79	Vac
LOC111011240	McSCPL28		+	462	51,115.8	5.79	Vac
LOC111011335	McSCPL29		+	433	48,114.7	6.27	Vac
LOC111011336	McSCPL30		+	433	47,813.4	7.86	Vac
LOC111019252	McSCPL31		+	1457	161,625.4	7.24	Vac
LOC111008438	McSCPL32		+	268	31,091.9	8.34	Vac

+: Confirmed in according databases. Vac: Vacuole; Pero: Peroxisome.

## Data Availability

The RNA-seq datasets of the tissue samples (ovary, male, flower, leaf, stem, root) are obtained from the SRA database (http://www.ncbi.nlm.nih.gov/sra/, accessed on 5 October 2023) through the accession numbers SRR13308550–SRR13308554 (PRJNA687997). The RNA-seq datasets of the fungal infection assays are available in the NCBI repository (http://www.ncbi.nlm.nih.gov/bioproject/PRJNA825666, accessed on 5 October 2023). The genome data of bitter gourd are available in the NCBI database (Assembly version: GCF_001995035.1; PRJNA397875).

## Supplementary Materials

The following supporting information can be downloaded at: https://www.mdpi.com/article/10.3390/ijms252111816/s1.

## Abbreviations

SCPL: serine carboxypeptidase-like; FOM: *Fusarium oxysporum* f. sp. *Momordicae*; SA: salicylic acid; JA: jasmonic acid; RNA-seq: RNA sequencing; FPKM: fragments per kilobase of transcript per million mapped reads; qRT-PCR: real-time quantitative reverse transcription polymerase chain reaction; SE: standard errors.
