# Supplementary figures and images for: Genome-Wide Analysis of the Serine Carboxypeptidase-like (SCPL) Protein Family of Bitter Gourd and Functional Validation of McSCPL22 in Fusarium oxysporum f. sp. Momordicae (FOM) Resistance
Source: Int J Mol Sci. 2024 Nov 3;25(21):11816. doi: 10.3390/ijms252111816 (PMC11546080; doi:10.3390/ijms252111816)

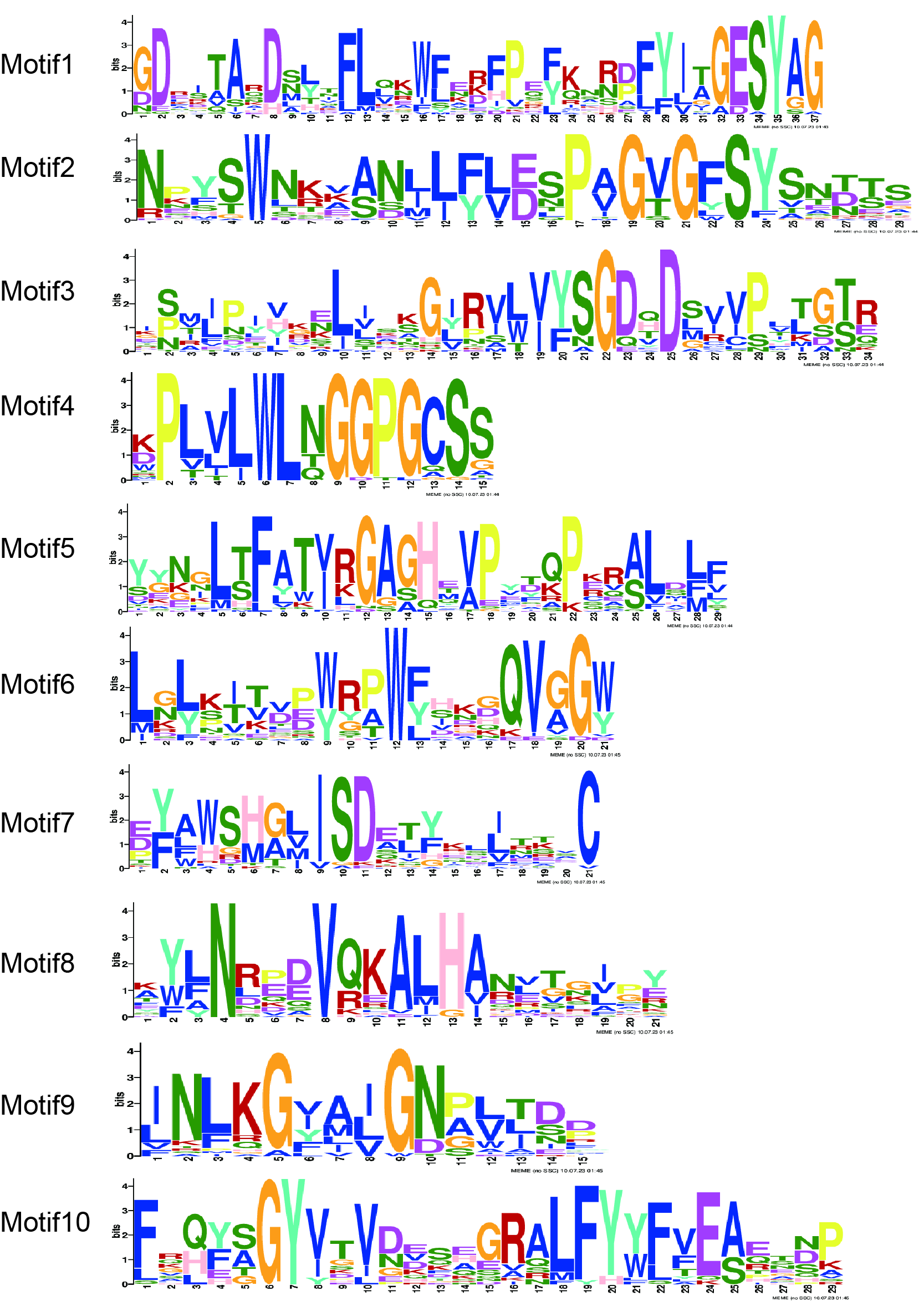

Supplement: Supplementary file 1 [file ijms-25-11816-s001.zip › Supplement Figure S1.tif]
